# FLASH protects ZEB1 from degradation and supports cancer cells' epithelial-to-mesenchymal transition

**DOI:** 10.1038/oncsis.2016.55

**Published:** 2016-08-15

**Authors:** C F Abshire, J L Carroll, A-M Dragoi

**Affiliations:** 1 Department of Medicine, Feist-Weiller Cancer Center, LSU Health, Shreveport, LA, USA

## Abstract

Cancer metastasis remains a significant challenge and the leading cause of cancer-associated deaths. It is postulated that during metastasis cells undergo epithelial-to-mesenchymal transition (EMT), a process characterized by loss of cell–cell contacts and increased migratory and invasive potential. ZEB1 is one the most prominent transcriptional repressors of genes associated with EMT. We identified caspase-8-associated protein 2 (CASP8AP2 or FLASH) as a novel posttranscriptional regulator of ZEB1. Here we demonstrate that FLASH protects ZEB1 from proteasomal degradation brought by the action of the ubiquitin ligases SIAH1 and F-box protein FBXO45. As a result, loss of FLASH rapidly destabilized ZEB1 and reversed EMT cellular characteristics. Importantly, loss of FLASH blocked transforming growth factor-β-induced EMT and enhanced sensitivity to chemotherapy. Thus, we propose that FLASH–ZEB1 interplay may be a protective mechanism against ZEB1 degradation in cells undergoing EMT and may be an efficacious target for therapies aimed to block EMT progression.

## Introduction

During metastasis cells undergo epithelial-to-mesenchymal transition (EMT), a process characterized by loss of cell polarity and cell–cell contacts and increased migratory and invasive potential.^1, 2^ EMT is triggered by a number of factors, including extracellular matrix components and growth factors, most notably transforming growth factor beta (TGFβ).^3, 4, 5^ Critical to EMT initiation is the gain of ZEB1/ZEB2, SNAIL/SLUG and TWIST1/2 transcriptional factors' expression and the functional loss of E-cadherin—a major component of the cell–cell junctions in epithelial cells.^6, 7, 8^ As an adherens junction component, E-cadherin acts as a tumor suppressor by contributing to epithelium integrity and by sequestering β-catenin, thereby restricting the mitogenic activity of β-catenin/T-cell factor pathway. ZEB1 is an essential EMT transcriptional activator and mediator of tumor radioresistance and chemoresistance.^9, 10, 11, 12^ Aberrant expression of ZEB1 has been documented in multiple cancers.^13^ Recently, the regulation of ZEB1 protein turnover has come into focus with the discovery of SIAH1/2 E3 ligases and Skp1-Pam-FBXO45 atypical ubiquitin E3 ligase complex as regulators of ZEB1 ubiquitination and degradation.^14, 15^

We have recently identified FLICE/caspase-8-associated huge protein (FLASH)/casp8ap2 as a repressor of E-cadherin expression through posttranscriptional control of ZEB1.^16^ Loss of FLASH specifically decreased ZEB1 protein expression in cancer cells resulting in de-repression of ZEB1-regulated genes involved in maintenance of the epithelial phenotype, such as E-cadherin. FLASH is involved in various cellular functions, including regulation of apoptosis, transcriptional regulation, regulation of replication-dependent histone gene expression and cell cycle progression.^17, 18, 19, 20^ Loss of FLASH expression has been shown to inhibit cell cycle progression in the S-phase in multiple cell lines owing to suppressed expression of histone genes.^19, 21^ Interestingly, of the 1982 amino acids of FLASH, only the first ~150 residues are required for histone pre-mRNA processing, whereas the remaining domains are involved in interactions with caspase-8,^22^ NPAT,^23^ c-myb^18^ and ZEB1.^16^ Although FLASH and ZEB1 can form a nuclear complex,^16^ it is unclear whether FLASH regulates EMT through modulating ZEB1 function or promoting ZEB1 stability. Whereas ZEB1 is widely accepted as one of the most important activators of EMT and recently revealed as a mediator of tumor radioresistance and drug resistance, the role of FLASH in solid tumors' growth and dissemination is unknown.

Here we expand on our earlier studies and show that the mechanism of FLASH-dependent control of ZEB1 function is conserved in multiple cancer cell lines, including cervical, breast, pancreas and prostate cancer, and it is dependent on ZEB1 proteasomal degradation. We also found that loss of FLASH led to ZEB1 ubiquitination by SIAH1 and FBXO45, resulting in ZEB1 degradation by the proteasome and EMT reversal. Importantly, loss of FLASH blocked initiation of EMT by TGFβ and reversed chemotherapy resistance in pancreatic cancer cells treated with gemcitabine. Overall our data identifies FLASH as an important EMT regulator that protects ZEB1 from degradation.

## Results

### FLASH controls ZEB1 and E-cadherin expression through a conserved mechanism

**Figure 1:**
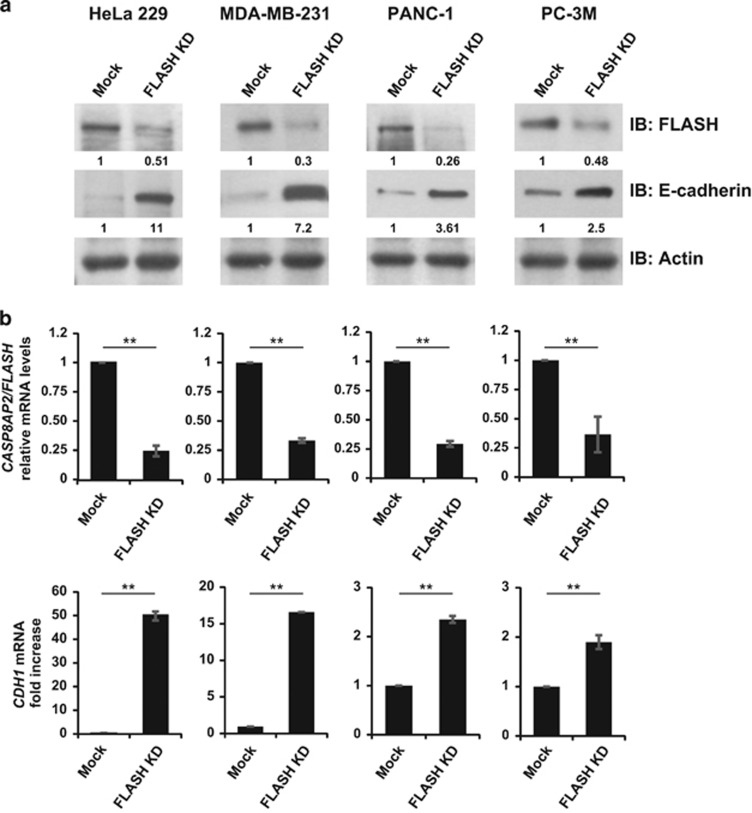
FLASH controls E-cadherin expression through a conserved mechanism in multiple cell lines. (**a**) HeLa 229, MDA-MB-231, PANC-1 and PC-3M cells were transfected with a pool of siRNA duplexes targeting FLASH. FLASH (top panel) and E-cadherin (middle panel) protein levels in siRNA- and mock-transfected cells were determined by western blot analysis and the expression normalized to actin loading control (bottom panel). (**b**) Relative mRNA levels of FLASH (top graphs) and E-cadherin (CDH1) (bottom graphs) as determined by quantitative PCR. The graphs represent the average of three independent experiments. The significance of differences was determined by Student's *t*-test (**P<0.0025).

**Figure 2:**
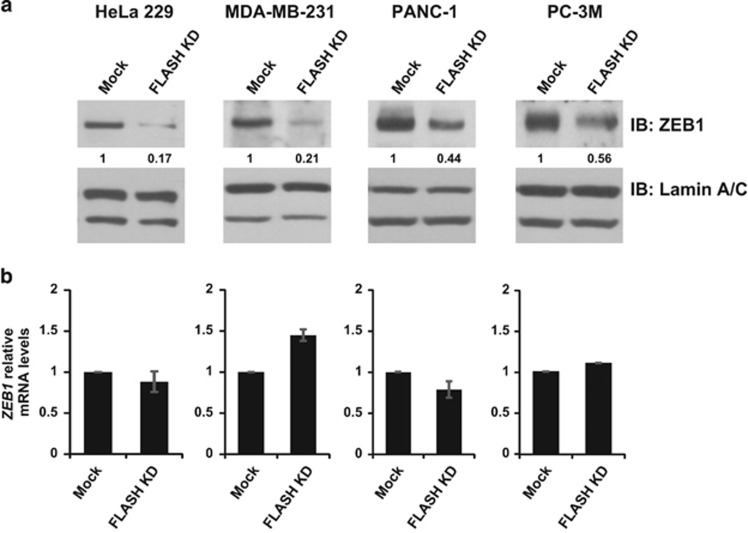
FLASH controls ZEB1 expression posttranscriptionally. (**a**) HeLa299, MDA-MB-231, PANC-1 and PC-3M cells were transfected with a pool of siRNA duplexes targeting FLASH and nuclear expression of ZEB1 was determined by western blot (top panel). ZEB1 expression was normalized to lamin A/C loading control (bottom panel). (**b**) Relative mRNA level of ZEB1 as determined by quantitative PCR.

Previously, we reported that loss of FLASH significantly upregulated E-cadherin (*CDH1*) gene expression in the cervical cancer line HeLa 229.^16^ To determine whether FLASH is a conserved regulator of E-cadherin in cancer cells, we silenced *CASP8AP2/FLASH* gene expression by siRNA duplexes in four distinct cell lines generated from diverse tissues. Depletion of FLASH in HeLa 229 (cervical cancer), MDA-MB-231 (triple-negative breast cancer), PANC-1 (pancreatic cancer) and PC-3M (prostate cancer) resulted in high expression of E-cadherin at the protein (Figure 1a, Mock vs FLASH KD) and mRNA level (Figure 1b, Mock vs FLASH KD). The loss of FLASH de-repressed E-cadherin expression in all four cell lines resulting in 2.5–11-fold increase in E-cadherin protein levels. In HeLa 229, loss of FLASH destabilizes ZEB1 resulting in E-cadherin upregulation.^16^ Therefore, we investigated whether loss of FLASH decreased the amount of ZEB1 protein. In all the cell lines tested, loss of FLASH reduced ZEB1 protein but not *ZEB1* mRNA (Figures 2a and b, Mock vs FLASH KD). Hence, the FLASH-ZEB1-E-cadherin axis we identified in HeLa 229 cells is a broadly conserved regulatory mechanism in multiple cancer cell lines originating from different organs.

### FLASH control of ZEB1 is independent of cell cycle arrest

To determine the mechanisms by which FLASH posttranscriptionally regulates ZEB1 function, we focused initially on the cell cycle because depletion of FLASH is known to arrest cells in S-phase.^19^ To this end we tested whether cell cycle arrest alone can recapitulate the effect of FLASH on ZEB1 and E-cadherin expression in cancer cells. To this end, we arrested cells in S-phase independent of FLASH by knocking down the methyltransferase SETD8—a known cell cycle progression regulator.^24^ Consistent with an S-phase cell cycle arrest phenotype, depletion of FLASH or SETD8 produced an equivalent increase in the percentage of cells in S-phase (Supplementary Figure S1A). Unlike FLASH-depleted cells, SETD8-depleted cells retained high levels of ZEB1 and low expression of E-cadherin despite being arrested in S-phase (Supplementary Figure S1B). Thus, FLASH regulation of ZEB1/E-cadherin is independent of FLASH function in cell cycle progression.

### FLASH regulates ZEB1 turnover by the proteasome

**Figure 3:**
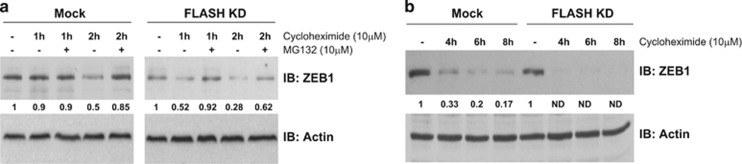
ZEB1 protein half-life is reduced in FLASH-depleted cells. (**a**) HeLa 229 cells were transfected with a pool of siRNA duplexes targeting FLASH and treated with cycloheximide alone (10 μm) or cycloheximide and MG132 (10 μm) for the indicated periods of time. ZEB1 expression was determined by western blot (top panel) and normalized to actin loading control (bottom panel). (**b**) Expression of ZEB1 after prolonged incubation with cycloheximide in FLASH-depleted and mock-transfected cells was determined by western blot analysis (top panel) and normalized to actin loading control (bottom panel).

Because FLASH and ZEB1 form a complex,^16^ next we investigated whether loss of FLASH reduced ZEB1 expression in cells owing to increased turnover brought by decreased ZEB1 stability. Thus, we determined the turnover rate of ZEB1 in mock and FLASH-depleted cells after protein synthesis was blocked with cycloheximide (Figure 3). In mock-treated HeLa 229 cells, ZEB1 half-life was 2 h, whereas in FLASH-depleted cells that rate decreased to 1 h (Figure 3a). After 4 h of cycloheximide treatment, ZEB1 was undetectable in FLASH-depleted cells, whereas 30% of ZEB1 remained in mock-transfected cells (Figure 3b). The increased ZEB1 turnover in the absence of FLASH was likely a direct result of accelerated proteasome-mediated degradation because the proteasome inhibitor MG132 restored ZEB1 protein levels (Figure 3a). These data indicate that loss of FLASH reduces the steady-state level of ZEB1 protein through an increase in ZEB1 proteasomal degradation.

### FLASH-induced ZEB1 degradation is dependent on the E3 ligases SIAH1 and FBXO45

**Figure 4:**
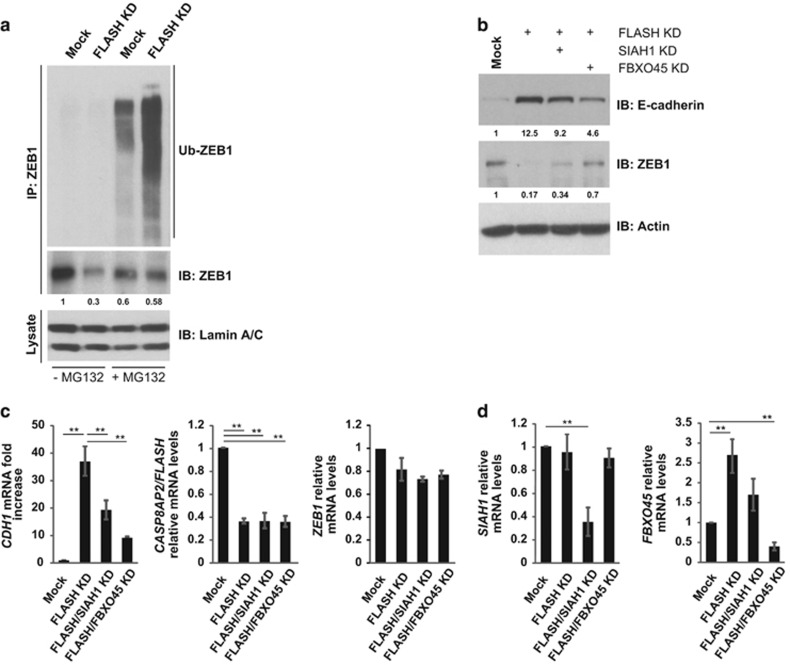
ZEB1 is degraded via the proteasomal pathway in FLASH-depleted cells. (**a**) HeLa 229 cells were transfected with a pool of siRNA duplexes targeting FLASH and treated with MG132 (10 μm) for 8 h. Nuclear extracts of siRNA- and mock-transfected cells were immunoprecipitated (IP) with anti-ZEB1 antibody and then immunoblotted for detection of ubiquitinated ZEB1 (Ub-ZEB1). Lamin A/C was used as a loading control (bottom panel). (**b**) E-cadherin (top panel) and ZEB1 (middle panel) expression in HeLa 229 cells following depletion of FLASH alone (FLASH KD), FLASH and SIAH1 (FLASH KD/SIAH1KD) and FLASH and FBXO45 (FLASH KD/FBXO45 KD). E-cadherin and ZEB1 expression was normalized to actin as loading control (bottom panel). (**c**) Relative mRNA levels of CDH1 (fold increase, left graph), FLASH (middle graph) and ZEB1 (right graph) as determined by quantitative PCR (qPCR). The graphs represent the average of three independent experiments. The significance of differences was determined by Student's *t*-test (**P<0.0025). (**d**) Relative mRNA levels of SIAH1 (left graph) and FBXO45 (right graph) as determined by qPCR. The graphs represent the average of three independent experiments. The significance of differences was determined by Student's *t*-test (**P<0.0025).

To investigate the ZEB1 proteasome-targeting mechanism, we examined ZEB1 ubiquitination. In HeLa 229 nuclear extracts, ZEB1 ubiquitination was undetectable unless the proteasome was inhibited with MG132, indicating ubiquitinated ZEB1 is degraded by the proteasome rapidly (Figure 4a). Although loss of FLASH decreased the amount of steady-state ZEB1 in the nucleus, ubiquitinated ZEB1 was undetectable unless the proteasome was blocked, which is consistent with the rapid turnover of ubiquitinated ZEB1. Importantly, the amount of steady-state ubiquitinated ZEB1 protected by MG132 drastically increased when FLASH was depleted from HeLa 229 cells indicating that absence of FLASH results in increased ZEB1 ubiquitination, which is subsequently degraded by the proteasome (Figure 4a, FLASH KD). The increase of total ZEB1 protein observed in FLASH-depleted cells treated with the MG132 (Figure 4a, FLASH KD+MG132) further supports a role for FLASH in ZEB1 protection from ubiquitination and proteasomal degradation.

To determine the regulators of ZEB1 turnover in FLASH-depleted cells, we focused on two ubiquitin ligases (UBLs) known to regulate ZEB1 steady-state amount in cells—SIAH1 and FBXO45.^14, 15^ Because MG132 rescues ZEB1 expression in the absence of FLASH, ablation of the ZEB1 UBLs should increase steady-state amount of ZEB1 in FLASH-depleted cells. Thus, we examined ZEB1 amount in HeLa 229 cells transfected with siRNAs for FLASH/SIAH1, FLASH/FBXO45 or FLASH alone. Loss of FBXO45 in FLASH-depleted cells drastically increased ZEB1 protein from 17% to 70% of the level of ZEB1 in cells expressing FLASH (Figure 4b, Mock vs FLASH KD vs FLASH/FBXO45 KD). Importantly, increased ZEB1 levels caused by FBXO45 depletion decreased E-cadherin protein and mRNA expression (*CDH1*), indicating that ZEB1 function is partially restored in FLASH/FBXO45-depleted cells (Figures 4b and c, Mock vs FLASH KD and Mock vs FLASH/FBXO45 KD). Similarly, loss of SIAH1 in FLASH-depleted cells increased ZEB1 but only from 17% to 34% of the ZEB1 level in cells expressing FLASH (Figure 4b, Mock vs FLASH KD vs FLASH/SIAH1 KD). We also examined ZEB1 and E-cadherin protein expression in cells expressing normal levels of FLASH but depleted for either SIAH1 or FBXO45. As expected, an increase in ZEB1 expression and a consequent decrease in E-cadherin levels was observed in cells lacking the UBLs (Supplementary Figure S2B). Because siRNAs against SIAH1 and FBXO45 specifically reduced their target mRNA levels by 70% and did not affect *ZEB1* or *CASP8AP2/FLASH* mRNA levels (Figures 4c and d), these data indicate that, although SIAH1 contributes to ZEB1 ubiquitination in FLASH-depleted cells, this process is mainly catalyzed by FBXO45. Moreover, loss of FLASH led to a significant increase in the *FBXO45* but not *SIAH1* mRNA, indicating that FLASH may regulate ZEB1 UBLs by multiple distinct mechanisms (Figure 4d).

### Loss of FLASH blocks EMT induced by TGFβ

**Figure 5:**
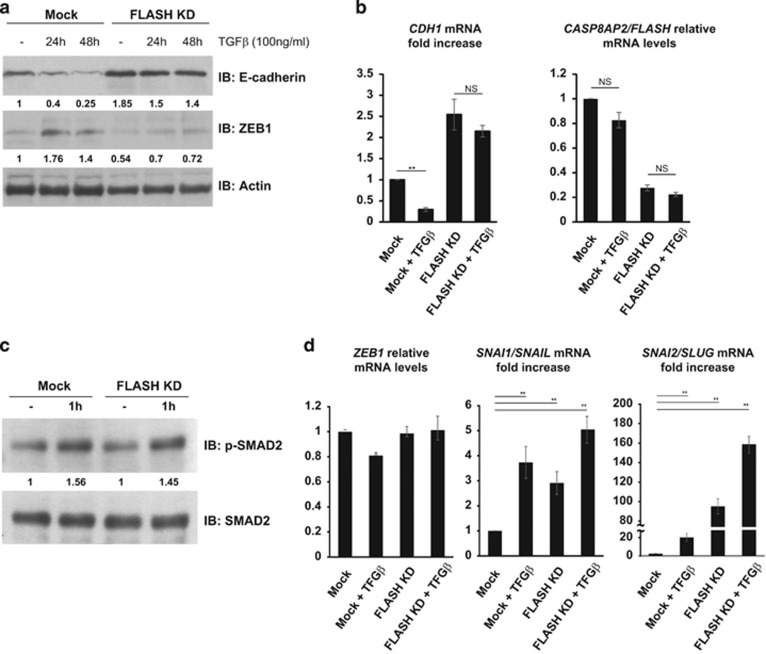
The role of FLASH in EMT progression. (**a**) PANC-1 cells were transfected with a pool of siRNA duplexes targeting FLASH and treated with 100 ng/ml TGFβ for the indicated periods of time. E-cadherin (top panel) and ZEB1 (middle panel) protein levels in siRNA- and mock-transfected cells treated with TGF-β or left untreated were determined by western blot analysis and the expression normalized to actin loading control (bottom panel). (**b**) Relative mRNA levels of E-cadherin (fold increase, left graph) and FLASH (right graph) were determined by quantitative PCR (qPCR). The graphs represent the average of three independent experiments. The significance of differences was determined by Student's *t*-test (**P<0.0025). (**c**) PANC-1 cells were transfected with a pool of siRNA duplexes targeting FLASH and treated with TGFβ (100 ng/ml) for 1 h. Phosphorylated SMAD2 and total SMAD2 protein levels in mock-transfected and FLASH-depleted cells treated with TGFβ was determined by western blot. (**d**) ZEB1, Snail and Slug mRNA expression in mock-transfected and FLASH-depleted cells as detected by qPCR. The graphs represent the average of three independent experiments. The significance of differences was determined by Student's *t*-test (**P<0.0025). NS, not significant.

To assess the physiological importance of FLASH in regulating EMT progression, we used TGFβ-induced EMT in the pancreatic adenocarcinoma cells PANC-1. TGFβ is a critical regulator of EMT in multiple cancers through mediation of transcriptional repression of genes associated with the epithelial phenotype.^3, 4^ Treatment of PANC-1 cells with TGFβ over 48 h period induced ZEB1 and drastically reduced the amount of E-cadherin protein and mRNA in cells (Figures 5a and b, Mock), confirming that TGFβ-treated cells undergo EMT. Conversely, FLASH-depleted PANC-1 cells failed to upregulate ZEB1 in response to TGFβ and as a consequence expressed E-cadherin protein and mRNA (*CDH1*) to high levels (Figures 5a and b, FLASH KD). In contrast, TGFβ treatment did not alter FLASH expression at either mRNA or protein level (Figure 5b and Supplementary Figure S3). Importantly, FLASH depletion did not perturb the ability of PANC-1 cells to respond to TGFβ because the amount of phosho-SMAD2 in TGFβ-treated cells was equivalent, irrespective of FLASH expression (Figure 5c). Thus, FLASH-depleted PANC-1 cells are refractory to TGFβ-induced loss of E-cadherin and retain an epithelial-like phenotype despite TGFβ stimulation.

Moreover, FLASH ablation interfered with TGFβ induction of ZEB1 protein without affecting *ZEB1* mRNA (Figure 5d), consistent with the idea of an increased ZEB1 turnover mediating resistance to TGFβ-induced EMT. Irrespective of FLASH expression in cells, TGFβ treatment significantly upregulated the mRNAs of two additional transcriptional repressors of E-cadherin—*SNAI1/SNAIL* and *SNAI2/SLUG* (Figure 5d). Regulation of SNAIL and SLUG by loss of FLASH alone and TGFβ treatment was also confirmed at the protein level by immunoblot analysis (Supplementary Figure S3). Thus, loss of FLASH does not affect TGFβ signaling but rather blocks TGFβ-induced EMT by decreasing the steady-state level of ZEB1, resulting in high E-cadherin expression despite the induction of the other transcriptional repressors SNAIL and SLUG.

**Figure 6:**
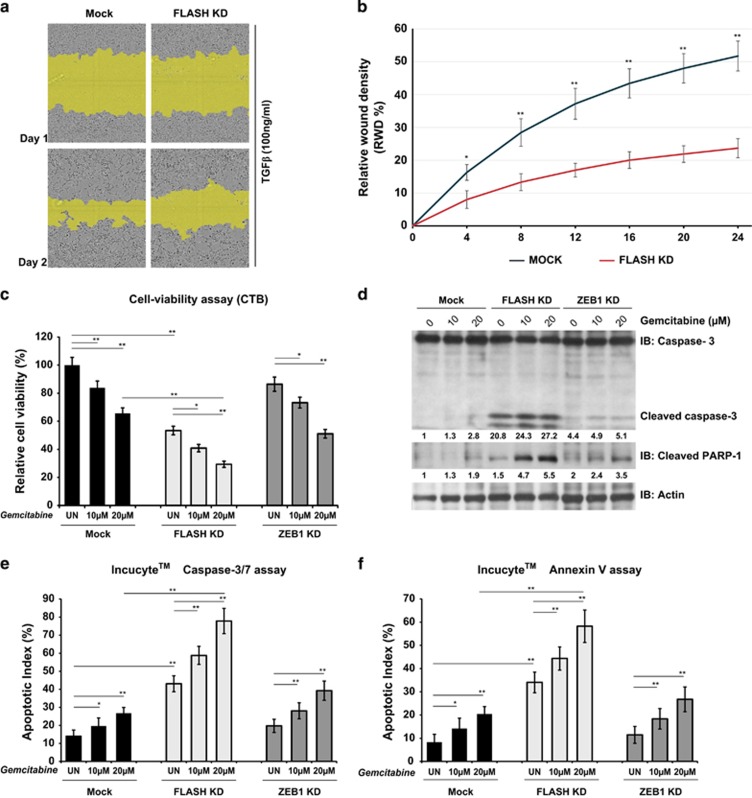
The role of FLASH in cell migration and chemoresistance. (**a**) Representative images of wound-healing assay in siRNA- and mock-transfected PANC-1 cells treated with 100 ng/ml TGFβ for 24 h. Images were acquired every 4 h for a total period of 24 h using IncuCyte ZOOM Live Cell Imaging system. The mask generated by the IncuCyte ZOOM analysis algorithm is shown in yellow. (**b**) Quantification of PANC-1 cells migration using the ‘Relative wound density (%)' metric module of the IncuCyte ZOOM software. The significance of differences was confirmed by Student's *t*-test (*P<0.005; **P<0.0025). (**c**) Cell viability of mock-transfected, FLASH-depleted and ZEB1-depleted cells left untreated or treated with gemcitabine was assayed 72 h posttreatment (CellTiter Blue assay). The graphs represent the average of three independent experiments. The significance of differences was determined by Student's *t-*test (*P<0.05, **P<0.0025). (**d**) Apoptosis initiation by gemcitabine treatment on FLASH-depleted or ZEB1-depleted PANC-1 cells was examined by western blot analysis of cleaved caspase-3 (top panel) and cleaved PARP-1 (middle panel) protein levels. Cleaved caspase-3 and cleaved PARP-1 levels were normalized to actin (bottom panel) as a loading control. (**e**, **f**) Induction of apoptosis in mock- and siRNA-transfected cells after treatment with gemcitabine. Cells transfected with the indicated siRNA-duplexes were treated with gemcitabine, and Incucyte Caspase-3/7 Green Reagent for Apoptosis (**e**) or Incucyte Annexin V Green Reagent for Apoptosis (**f**) were added simultaneously. Treatments were carried out in triplicate. Green fluorescent confluence was normalized to cell total confluence to generate the Apoptotic Index (%). The graphs represent the average of three independent experiments. The significance of differences was determined by Student's *t*-test (*P<0.05, **P<0.0025).

To determine whether FLASH depletion blocked TGFβ-mediated progression toward a mesenchymal phenotype characterized by high motility and invasiveness, we measured cell migration. In a wound-healing assay, FLASH-depleted PANC-1 cells migrated significantly slower as compared with a mock-transfected control following TGFβ treatment (Figures 6a and b), lending additional support to the idea that loss of FLASH enforces an epithelial phenotype.

### Loss of FLASH increases sensitivity to gemcitabine treatment

Another critical outcome of high levels of ZEB1 in cancer progression is induced resistance to chemotherapy.^12^ Therefore, we investigated whether FLASH depletion can sensitize cancer cells to chemotherapy. To this end, mock- and siRNA-transfected PANC-1 cells were treated with sublethal doses of gemcitabine for 72 h, and cell viability was measured by CellTiter Blue assay (CTB). Gemcitabine treatment reduced cell viability in mock-transfected and FLASH- or ZEB1-depleted cells (Figure 6c). Interestingly, loss of FLASH alone resulted in a 45% reduction in cells' viability (Figure 6c, FLASH KD). Treatment with gemcitabine further reduced cell viability by 75% in FLASH-depleted cells, significantly farther than gemcitabine treatment in mock-transfected cells, suggesting that FLASH expression can promote chemoresistance. We also evaluated cell apoptosis by caspase-3 and PARP-1 (poly ADP-ribose polymerase 1) cleavage (Figure 6d). Under these conditions, minor apoptotic cell death was detected in mock-transfected cells at high levels of gemcitabine treatment (Figure 6d, Mock). However, gemcitabine treatment induced apoptosis in cells transfected with either FLASH or ZEB1 siRNA in a dose-dependent manner (Figure 6d, FLASH KD and ZEB1 KD). In order to quantify the cell death response induced by gemcitabine treatment, apoptotic cells were analyzed for Caspase-3/7 activation (Figure 6e) and Annexin V binding (Figure 6f). Consistent with our cell viability assay, loss of FLASH alone was sufficient to induce apoptosis in pancreatic cancer cells, which was further increased by gemcitabine treatment (Figures 6e and f, FLASH KD vs Mock). Interestingly, loss of FLASH had a far greater impact on cell survival as compared with loss of ZEB1, suggesting that additional factors controlled by FLASH contribute to chemotherapy resistance (Figures 6c–f, FLASH KD vs ZEB1 KD). These data further support a role of FLASH as a pro-survival factor as has been reported in specific contexts in cell lines derived from solid tumors.^20, 25^ Thus, development of small molecules to block FLASH function in solid tumors could benefit cancer therapy in several ways by reversing chemoresistance, interfering with metastasis and blocking cancer cell proliferation.

## Discussion

Our original investigation into the regulatory network of E-cadherin transcriptional repression revealed that loss of FLASH restores high levels of E-cadherin in cancer cells.^16^ Moreover, we found that FLASH is regulating ZEB1 protein expression while *ZEB1* mRNA level remained largely unchanged. Reduced ZEB1 expression in cancer cells resulted in de-repression of multiple ZEB1-regulated genes involved in maintenance of the epithelial phenotype.

Here we extended those studies to demonstrate that FLASH protects ZEB1 from proteasomal degradation through a mechanism conserved in multiple cancer cell lines. We provide evidence that ZEB1 protein half-life is drastically reduced in FLASH-depleted cells (Figure 3) while *ZEB1* mRNA levels are unchanged under those conditions (Figure 2b). We demonstrate that the FLASH-dependent ZEB1 degradation requires the ubiquitin–proteasome system and functions through SIAH1 E3 ubiquitin ligase and the F-box protein FBXO45 atypical E3 ligase. Blocking the proteasome or depleting the ZEB1 UBLs restored the amount of functional ZEB1 protein, which in turn, reduced E-cadherin levels (Figures 4a and b). Furthermore, depletion of SIAH1 or FBXO45 in cells expressing normal levels of FLASH resulted in increased ZEB1 protein expression (Supplementary Figure S2B).

These results demonstrate that regulators of ZEB1 protein stability are critical for ZEB1 function in cancer cells. Thus, ZEB1 protein expression is controlled through a complex and yet to be fully understood mechanism. Previous studies show that mRNA levels of various EMT transcription factors, including *ZEB1*, were not significantly different among normal tissues and tumor tissues.^15^ Yet, malignant tumors frequently displayed high protein expression of the EMT transcription factors in the absence of overt mRNA changes.^15^ These findings support the idea that posttranscriptional mechanisms, such as the ones regulating protein stability and degradation, could maintain high levels of the EMT transcription factors in tumor cells. Because these processes might be selectively deregulated in cancer cells during EMT progression, ZEB1 stability and its regulators represent attractive targets for development of therapeutic interventions.

Because FLASH and ZEB1 form a complex,^16^ FLASH could regulate ZEB1 stability through one or more potential mechanisms. On one hand, FLASH might conceal the UBL recognition sites on ZEB1 or compete with the ZEB1 UBLs for binding through its direct interaction with ZEB1. Alternatively, FLASH could repress the transcription of ZEB1 UBL genes. We found that FLASH regulates FBXO45 but not SIAH1 gene expression (Figure 4d), although ablation of either of the UBLs partially restored ZEB1 protein expression in FLASH-depleted cells (Figure 4b). Additionally we observe that loss of SIAH1 also results in a reduction of FBXO45 expression in cells deficient for FLASH but not in cells expressing normal levels of FLASH (Figure 4d and Supplementary Figure S2A). This suggests additional layers of regulation of ZEB1 UBLs in FLASH-depleted cells. The extent of direct or indirect effect of SIAH1 on ZEB1 degradation in FLASH-depleted cells therefore remains to be determined. Because both SIAH1 and FBXO45 are involved in ZEB1 stability and possibly act redundantly, we speculate that multiple ubiquitin ligases might contribute to ZEB1 ubiquitination and proteasomal degradation. Hence, FLASH likely maintains ZEB1 stability through distinct mechanisms controlled by different ZEB1 UBLs.

The TGFβ pathway is known to be the primary inducer of EMT through increased expression of E-cadherin repressors and EMT regulators, such as ZEB1, SNAIL and SLUG.^3, 4^ The physiological relevance of ZEB1 protection by FLASH was demonstrated by the requirement of FLASH for TGFβ-induced EMT in PANC-1 cells. FLASH depletion prevented E-cadherin loss in cells treated with TGFβ despite a functional TGFβ pathway and a significant increase in *SNAI1/SNAIL* and *SNAI2/SLUG* mRNAs (Figures 5a and d). Thus, in PANC-1 cells, the FLASH-ZEB1 pathway can override the functions of the other central E-cadherin repressors SNAIL and SLUG. Alternatively, FLASH might be directly involved in SNAIL and SLUG transcriptional repression of E-cadherin or indirectly through regulation of additional co-factors required for E-cadherin repression by SNAIL family. It is known that ZEB1, SNAIL and SLUG require a different set of co-factors in order to exert their repression function on E-cadherin.^13^ Thus, the correlation between expression of SNAIL or SLUG and E-cadherin repression in FLASH-depleted cells could be determined by the FLASH effect on the interacting co-factors. It is also possible that, despite phosphorylation of SMAD proteins and initial activation of the TGFβ signaling pathway, absence of FLASH limits TGFβ effects owing to altered expression and/or interference with downstream regulators required for optimal TGFβ responses.

An essential element of EMT progression is induction of chemotherapy resistance.^12, 26, 27^ In pancreatic cells, ZEB1 expression level correlates with chemotherapy resistance to gemcitabine, 5-fluorouracil and cisplatin.^12^ In this study, we uncovered that loss of FLASH or loss of ZEB1 antagonized gemcitabine resistance in PANC-1 cancer cells. Cells lacking either FLASH or ZEB1 exhibited increased sensitivity to gemcitabine and initiated apoptosis at low doses of gemcitabine (Figures 6c–f). Interestingly, loss of FLASH sensitized cells to gemcitabine more than ZEB1 loss and was sufficient to promote apoptosis even in the absence of treatment. Thus, FLASH might contribute to chemoresistance through ZEB1-dependent and -independent mechanisms.

FLASH was originally identified as a pro-apoptotic protein involved in Fas-mediated caspase-8 activation.^22^ In acute lymphoblastic leukemia patients, loss of FLASH expression was correlated with poor treatment response and relapse.^28, 29^ However, an antiapoptotic role for FLASH has been described in studies showing that FLASH can suppress apoptosis in both Fas-dependent and -independent manners.^20, 25^ These studies performed in fibrosarcoma cells and colorectal carcinoma cells suggest that FLASH can promote or inhibit apoptosis depending on the context and cell type involved. In our study, loss of FLASH in pancreatic cells resulted in (1) reduced cell viability in the absence of chemotherapeutic treatment, (2) increased degradation of PARP-1 and (3) higher apoptotic rates in cells treated with low doses of gemcitabine. Our observation that loss of FLASH and gemcitabine treatment synergized to promote PANC-1 cell death suggests that FLASH employs multiple mechanisms to promote cancer cell survival: (1) as a pro-survival factor and (2) as a chemoresistance factor.

Whether the distinct FLASH loss-of-function phenotypes in solid tumors and blood tumors are due to cell-type specific differences in FLASH-regulated transcriptional programs or FLASH-dependent regulation of apoptosis is of major interest. Because *FLASH* mRNA expression in primary tumors and colorectal cancer cell lines is similar to normal tissues,^20^ it is likely that mechanisms controlling FLASH function or protein levels in cells impact tumorigenesis. Therefore, studies that investigate FLASH function and the principal downstream effects associated with loss of FLASH function in various cell lines could elucidate the roles of FLASH in EMT and cancer progression. Considering the importance of ZEB1 in EMT initiation and chemoresistance, our study indicates that FLASH-mediated ZEB1 regulation could be exploited to design therapies directed toward ZEB1 degradation in cells undergoing EMT to block this key step in cancer progression.

## Materials and methods

### Cell culture conditions

HeLa 229 (CCL-2.1), MDA-MB-231 (HTB-26) and PANC-1 (CRL-1469) cells were obtained from ATCC (Manassas, VA, USA) (CCL-2.1, HTB-26, CRL-1469) and grown in Dulbecco's modified Eagle's medium (Sigma-Aldrich, St Louis, MO, USA) supplemented with 10% fetal bovine serum. PC-3M cells, a highly metastatic and well-characterized variant of the parental PC-3 cell line (CRL-1435), were a gift from Dr James Cardelli and were grown in RPMI (Sigma-Aldrich) supplemented with 10% fetal bovine serum. Cells were cultured at 37 °C in a 5% CO_2_ incubator. All cell lines were authenticated by short tandem repeat profiling. All cell lines were tested for *Mycoplasma* contamination.

### RNAi assays

Cells were reverse transfected with Dharmafect1 (Dharmacon, Lafayette, CO, USA) and a pool of the four individual siRNA-silencing reagents (12.5 nmol/l each, 50 nmol/l total). For western blot analysis, FACS analysis and immunoprecipitation assay cells were transfected in 24- and 6-well plates, respectively, for 72 h. For real-time quantitative PCR analysis, cells were transfected in 96-well plates for 72 h. All siRNA duplexes were purchased from Dharmacon.

### Quantitative PCR

Total RNA and first-strand cDNA synthesis was performed using the TaqMan Gene Expression Cells-To-Ct Kit (ThermoFisher, Waltham, MA, USA) as recommended by the manufacturer. mRNA levels were determined by quantitative real-time PCR using the Universal ProbeLibrary (Roche Life Science, Indianapolis, IN, USA) and LightCycler 480 Probes Master (Roche Life Science). Thermal cycling was carried out using a LightCycler 96 instrument (Roche Diagnostics, Indianapolis, IN, USA) under the following conditions: 95 °C for 5 min and 45 cycles at 95 °C for 10 s and 60 °C for 25 s. Gene expression was normalized to glyceraldehyde 3-phosphate dehydrogenase. Efficiency of knockdown is represented as a decrease comparative to mock-transfected cells (value of 1). The fold increase is represented as relative values to the mock-transfected cells (value of 1).

### Immunoblotting and immunoprecipitation

HeLa 229, MDA-MB-231, PANC-1 and PC-3M cells grown for 3 days after siRNA transfection were lysed in dithiothreitol buffer prior to sodium dodecyl sulfate–polyacrylamide gel electrophoresis analysis and immunoblotting. For immunoprecipitation of ZEB1, nuclear fractions were prepared using the NE-PER Nuclear and Cytoplasmic Extraction Reagents (ThermoFisher). Immunoprecipitation was carried out in nuclear fractions appropriately diluted to reduce NaCl concentration with anti-ZEB1 antibody (H-102; Santa Cruz Biotechnology Inc, Dallas, TX, USA) and protein G-sepharose beads (GE Healthcare, Pittsburgh, PA, USA). Around 5% of the nuclear extracts were loaded for input control.

### Antibodies and reagents

The primary antibodies used anti-E-cadherin (Clone 36; BD Transduction Laboratories, San Jose, CA, USA), anti-ZEB1 (H-102; Santa Cruz), anti-actin (C-2; Santa Cruz), anti-FLASH (M-300; Santa Cruz), anti-caspase-3 (8G10; Cell Signaling Technology Inc, Danvers, MA, USA), anti-cleaved PARP-1 (D214; Cell Signaling), anti-lamin A/C (4C11; Cell Signaling), anti-ubiquitin (P4D1; Cell Signaling), anti-phospho-SMAD2 (D27F4; Cell Signaling), anti-SMAD2/3 (D7G7; Cell Signaling), anti-SNAIL (C15D3; Cell Signaling) and anti-SLUG (C19G7; Cell Signaling) were obtained commercially. Secondary antibodies horseradish peroxidase-conjugated anti-mouse and anti-rabbit (1:5000) were from Jackson Laboratories (West Grove, PA, USA). Gemcitabine was purchased from Tocris (Bristol, UK). MG132 and TGFβ were purchased from Cell Signaling.

### Wound-healing assay

For scratch wound-migration studies, PANC-1 cells were seeded at 10,000 cells/well and treated with TGFβ (100 ng/ml). The WoundMaker-96 tool from Essen Bioscience (Ann Arbor, MI, USA) was used to create identical wound in all wells. Images were acquired every 4 h for a 24 h period of time using IncuCyte ZOOM microscope (Essen Bioscience). Cell Migration software module was used for image analysis and Relative wound density (RWD %) metric was used to calculate the rate of migration.

### Cell viability assay

To determine cell viability, CellTiter Blue assay from Promega (Madison, WI, USA) was used. Shortly, 50 μl of PANC-1 cells were plated out in 96-well plates in triplicate and left overnight to adhere. Twenty-four hours later, cells were treated with gemcitabine as indicated. Treated cells were incubated for 72 h at 37 °C in 5% CO_2_. Later, the CellTiter Blue assay was performed according to the manufacturer's protocol and the fluorescence signal of resorufin was detected at 590 nm using a plate reader (Synergy 4, BioTek, Winooski, VT, USA). The relative viability of siRNA-transfected and gemcitabine-treated cells was determined after normalization to the average viability of mock-transfected untreated cells.

### Apoptosis assay

Apoptosis measurements in mock-treated and FLASH or ZEB1-depleted cells were performed on the IncuCyte ZOOM (Essen BioSciences) with a DEVD substrate (IncuCyte Caspase-3/7 Green Reagent for Apoptosis, Essen BioScience) and a phosphatidylserine-binding reagent (IncuCyte Annexin V Green Reagent for Apoptosis, Essen BioScience). Forty-eight hours post-siRNA treatment, cells were treated with gemcitabine in 96-well plates in triplicate and Caspase-3/7 reagent or Annexin V reagent was added to the cells. Phase-contrast and fluorescent images were acquired 72 h later. IncuCyte imaging software was used to calculate the confluency of fluorescent objects (%) per well as a measure of apoptosis. This value was normalized to the total cell confluency (%) of the same area of the image (phase contrast) to generate the apoptotic index for the individual treatments.

### Statistical analysis

All experiments were performed in triplicate. Data are presented as mean±s.d. Analysis utilized Student's *t*-tests to determine significance. Values of *P*<0.05 were considered significant, values of *P*<0.0025 were considered highly significant.
