# Supplementary material for: FLASH protects ZEB1 from degradation and supports cancer cells' epithelial-to-mesenchymal transition
Source: Oncogenesis. 2016 Aug 15;5(8):e254–. doi: 10.1038/oncsis.2016.55 (PMC5007829; doi:10.1038/oncsis.2016.55)
Supplement: Supplementary Information [file oncsis201655x1.pdf]

SUPPLEMENTARY FIGURE LEGENDS

Supplementary Figure 1. FLASH-dependent E-cadherin and ZEB1 regulation is independent of cell cycle arrest. A, HeLa 229 cells were transfected with a pool of siRNA duplexes targeting FLASH or SETD8. Flow cytometry cell cycle analysis was performed using propidium iodide (PI) DNA staining. **B**, E-cadherin (top panel) and ZEB1 (middle panel) protein levels as detected by Western blot analysis in FLASH-depleted and ZEB1-depleted HeLa 229 cells. Actin was used as a loading control (bottom panel).

Supplementary Figure 2. FBXO45 and SIAH1 are UBLs for ZEB1. HeLa 229 cells were transfected with a pool of siRNA duplexes targeting FBXO45 or SIAH1. **A,** FBXO45 and SIAH1 mRNA expression in mock-transfected and siRNA-transfected cells as detected by qPCR. The graphs represent the average of three independent experiments. **B**, ZEB1 (top panel) and E-cadherin (middle panel) protein levels in siRNA- and mock-transfected cells were determined by Western blot analysis and the expression normalized to actin loading control (bottom panel).

Supplementary Figure 3. FLASH regulates SNAIL and SLUG expression. PANC-1 cells were transfected with a pool of siRNA duplexes targeting FLASH and treated with 100 ng/ml TGF-β for the 48h. FLASH, E-cadherin, SLUG and SNAIL protein levels in siRNA- and mock-transfected cells treated with TGF-β or left untreated were determined by Western blot analysis and the expression normalized to actin loading control (bottom panel).

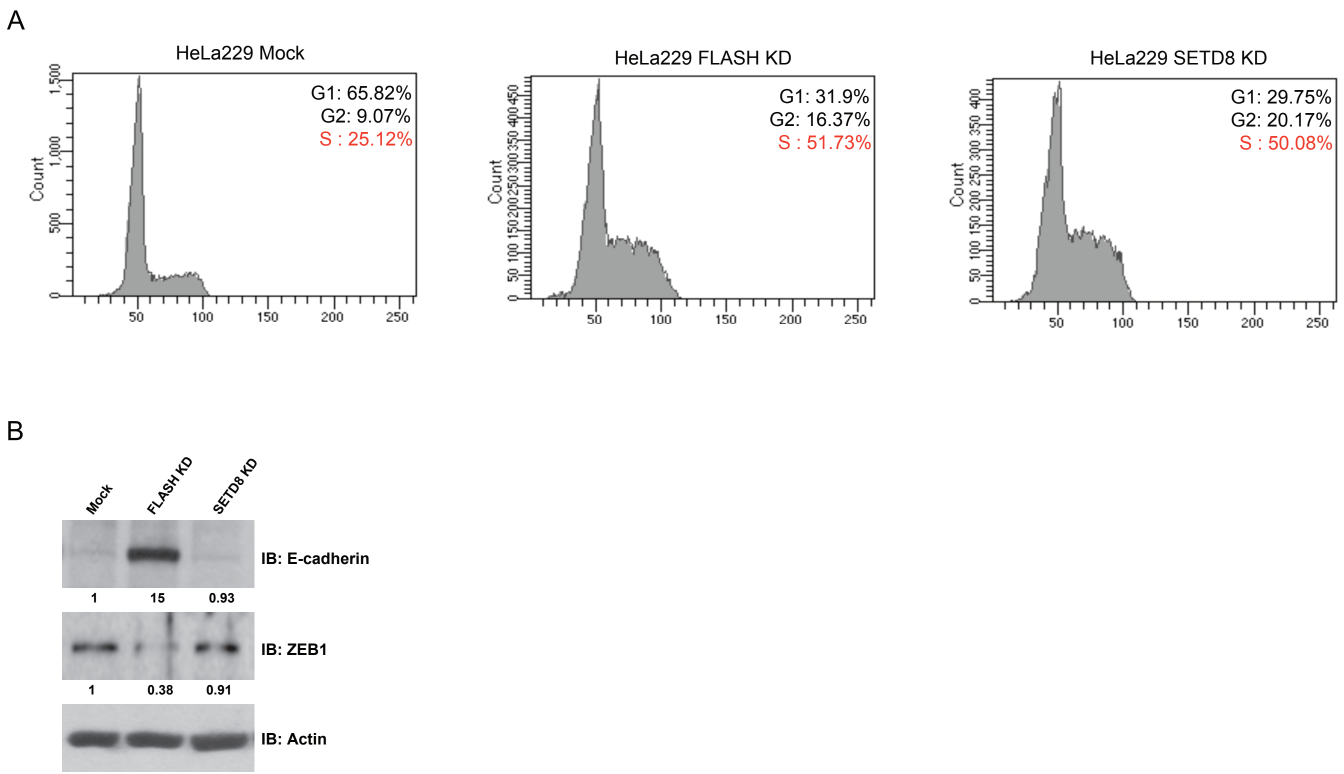

Supplementary Figure 1

A

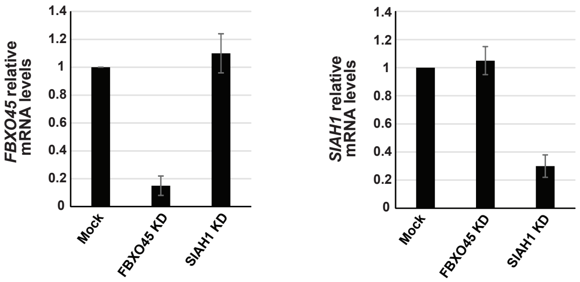

B

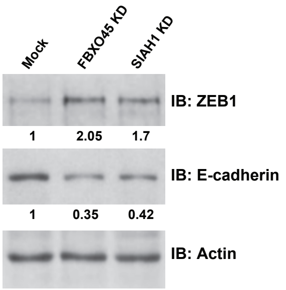

Supplementary Figure 2

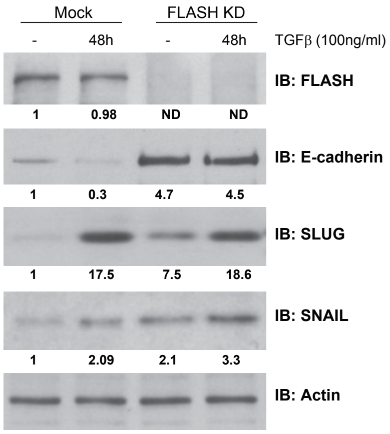

Supplementary Figure 3
